# Effect of Stiffening the Printed Circuit Board in the Fatigue Life of the Solder Joint

**DOI:** 10.3390/ma15186208

**Published:** 2022-09-07

**Authors:** Sushil Doranga, Matthew Schuldt, Mukunda Khanal

**Affiliations:** Department of Mechanical Engineering, Lamar University, Beaumont, TX 77710, USA

**Keywords:** resonance test, fatigue, solder joint, board thickness

## Abstract

Predictive analysis of the life of an electronic package requires a sequence of processes involving: (i) development of a finite element (FE) model, (ii) correlation of the FE model using experimental data, and (iii) development of a local model using the correlated FE model. The life of the critical components is obtained from the local model and is usually compared to the experimental results. Although the specifics of such analyses are available in the literature, a comparison among them and against the same electronic package with different user printed circuit board (PCB) thicknesses does not exist. This study addresses the issues raised during the design phase/life analysis, by considering a particular package with a variable geometric thickness of the user PCB. In this paper, the effect of stiffening the user PCB on the fatigue life of a ball grid array (BGA), SAC305 solder joint is studied. The board stiffness was varied by changing the thickness of the PCB, while the size of the substrate, chips, and solder balls were kept constant. The test vehicle consisted of BGA chips soldered to a user PCB. The thickness of the user PCB was varied, but the surface area of the BGA chip remained identical. The test vehicle was then modeled using a finite element analysis tool (ANSYS). Using a global/local modeling approach, the modal parameters in the simulations were correlated with experimental data. The first resonance frequency dwell test was carried out in ANSYS, and the high-cycle fatigue life was estimated using the stress-life approach. Following the simulation, the test vehicle was subjected to resonance fatigue testing by exciting at the first mode resonance frequency, the mode with the most severe solder joint failure. The resistance of the solder joint during the experiment was monitored using a daisy-chain circuit, and the point of failure was further confirmed using the destructive evaluation technique. Both the experimental and simulation results showed that stiffening the board will significantly increase the fatigue life of the solder joint. Although the amplitude of the acceleration response of the test vehicle will be higher due to board stiffening, the increase in natural frequencies will significantly reduce the amplitude of relative displacement between the PCB and the substrate.

## 1. Introduction

Vibration and thermal-based environmental loading are commonly encountered during the service life of electronic components, subsystems, and assemblies. However, compared to thermal loading, vibration-based loading is more complex and has long been the subject of research. In electronic manufacturing industries, virtually all the equipment commercially manufactured today uses surface mount technology, as this allows for far more components to be packed into a much smaller space. In surface mount technology, solder joints are the primary mechanical, thermal, and electrical interconnects between the components and the printed circuit board. Therefore, the reliability of solder joints is crucial in electronic packaging. A solder joint is a metallurgical joining method in which two or more metals are joined by melting and flowing a filler metal, commonly known as solder, into the joint. The traditional lead-containing solder alloy was banned due to the inherent toxicity of lead, and as a result, researchers around the world have focused on the development of a lead-free alternative. Recent advancements in lead-free solder alloys can be found in the technical literature [1,2,3]. With regards to the vibration-based reliability modeling of lead-free solder alloys, most of the research has focused on developing a general methodology to predict the fatigue life of solder joints. These general methods [4,5,6,7,8,9,10,11,12,13,14,15] include: (i) modal parameter estimation of the electronic package using the experimental modal analysis technique and updating the modal parameters in a finite element (FE) based model [4,5,6,7], (ii) detailed finite element modeling of the solder joint [8,9,10], (iii) calculation of the fatigue strength coefficient and exponent by combining the experimental and finite element model [11,12,15], (iv) utilizing the finite element-based simulation approach to obtain the critical stress in the solder ball, (v) calculating the fatigue life of the solder ball based on the maximum von Mises stress in the solder ball, and (vi) comparing the theoretical finite element results with the experiments. All of these general methods utilize frequency domain techniques for the harmonic vibration excitation, and the time domain-based rainflow cycle counting algorithm for the random vibration base excitation. Although significant progress has been made in developing a general method to evaluate the fatigue life of solder joints, there is a lack of understanding of the effect that specific properties of the PCB have on fatigue life. The properties of the user PCB include: (i) stiffness, (ii) dimensions, (iii) mode of vibration, and (iv) input excitation frequency of the PCB.

**Figure 1:**
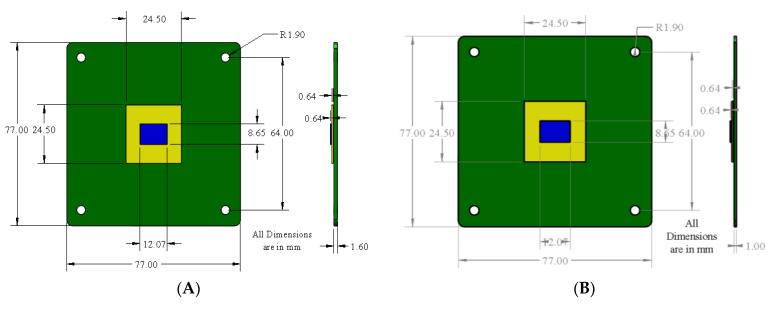

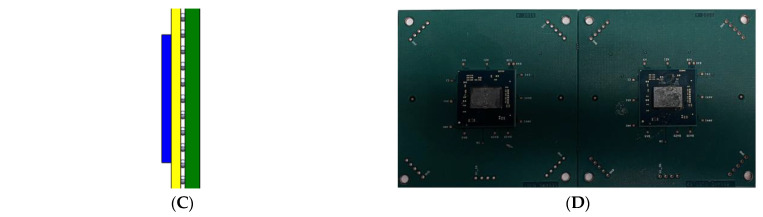
Test vehicle with different user PCB thicknesses (**A**): 1.6-mm board, (**B**): 1-mm board, (**C**): solder detail, (**D**) photograph of the test vehicle.

**Figure 2:**
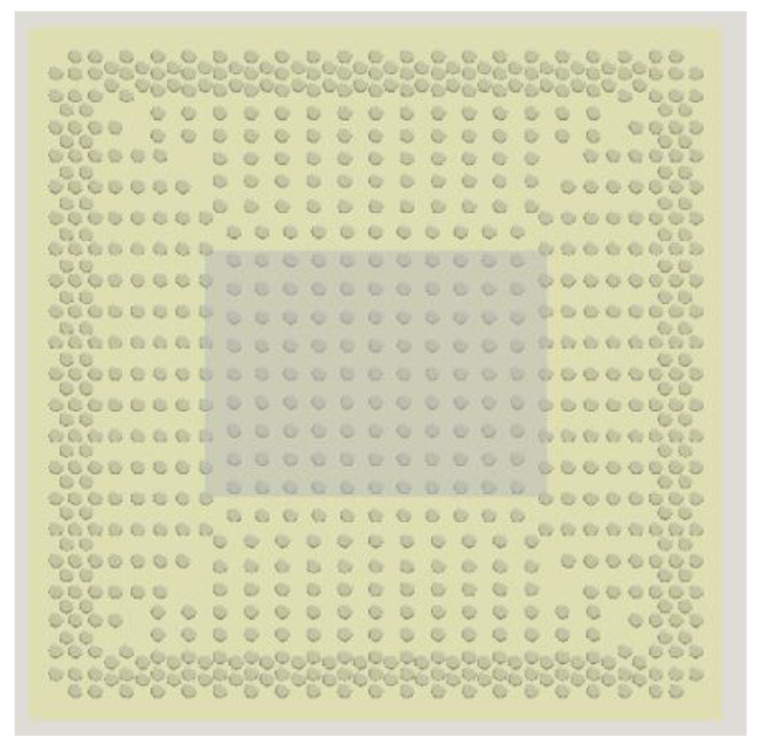
Detail of the solder ball (SAC 305).

In this paper, the worst-case vibration scenario (resonance-based fatigue) in a ball grid array (BGA) chip solder joint is studied using the finite element method and direct experimentation. The effect of increasing the PCB thickness on the overall life of the solder ball was investigated through experimentation and numerical simulation. The study was carried out in the first bending mode of the user PCB; the worst stress amplitude that the test vehicle will see under vibration. The test vehicle consisted of electronic chips and substrate mounted onto a user PCB using the BGA solder. The solder balls were electrically connected through daisy-chain circuits. The dimensions of the user PCBs were (77 mm × 77 mm × 1 mm) and (77 mm × 77 mm × 1.6 mm), where 1 and 1.6 mm are the thickness of the PCBs used, and 77 mm is the length and the width of the PCB. Figure 1 Shows the PCB with the chips, and Figure 2 gives a detailed view of the solder ball arrangement. As shown in Figure 1, two test vehicles are shown with PCBs of thickness of 1 mm and 1.6 mm. The solder balls electronics shown in Figure 2 were made of lead-free (SAC 305) tin/silver/copper alloys that contain 96.5% tin (Sn), 3% silver (Ag), and 0.5% copper (Cu). As BGA chips are used in different applications, including automotive, robo-taxi, locomotive, and aerospace industries, and the minimum PCB thickness requirement is different for each application, it is crucial to analyze the effect of board stiffening in the life of the solder joint.

The rest of the paper is organized as follows: Section 2 presents the methods and experiments, including the finite element analysis approach of the two test vehicles shown in Figure 1 and the experimental setup. Section 3 presents detailed information on the FE modeling results and the correlation between the experimental and the simulation results. Section 4 of the paper presents the conclusions of the work. 

## 2. Methods and Experiments

In this section, the detailed finite element analysis and experimental setup for the test vehicle are presented. The finite element model for a test vehicle was developed using the global/local modeling technique. The acceleration response data, modal damping ratios, and the natural frequencies of the test vehicle obtained from the finite element model were correlated with the experimental data. The correlated model was then utilized further for local analysis.

### 2.1. Finite Element Model

**Figure 3:**
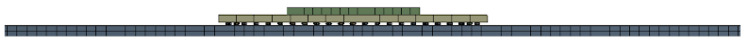
Global finite element model.

**Figure 4:**
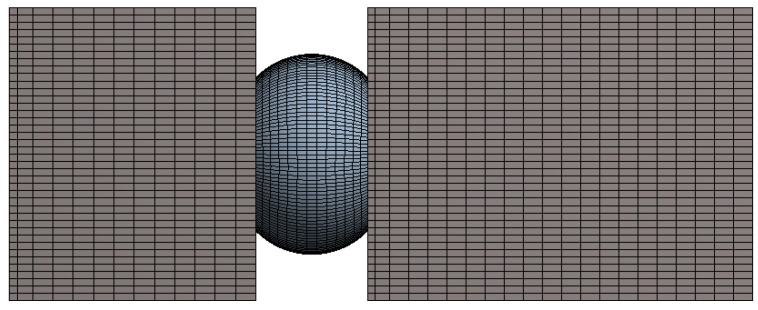
Local cut model after meshing.

The finite element model of the test vehicle was developed using the ANSYS structural analysis tool. The model consisted of a PCB, substrate, chip, and solder balls. There were a total of 769 solder balls in the assembly, as shown in Figure 2. It is well known that the stress induced in the inner balls is at its minimum during vibration loading [16,17,18] and can be neglected. Therefore, to reduce the complexity of the model, only the outer rows and columns were modeled. The chip (silicon die) was modeled above the substrate and the substrate and the chip, substrate and solder balls, and the solder ball and PCB were bonded. The system was modeled using 186 solids with quadratic elements. Figure 3 shows the global FE model of the test vehicle. A cut model was developed by taking one of the corner solder balls to create a local finite element model. It should be noted that while creating a cut model, the boundaries for the local model needed to be verified to ensure that they were far enough from the stress concentration region. Figure 4 shows the local cut model of the test vehicle. In order to analyze the cut model, displacement boundary conditions were used. These displacements were obtained by solving the global model. The local model was developed using the node sharing technique. The material properties that were used in the finite element analysis are tabulated in Table 1.

### 2.2. Experimental Setup

**Figure 5:**
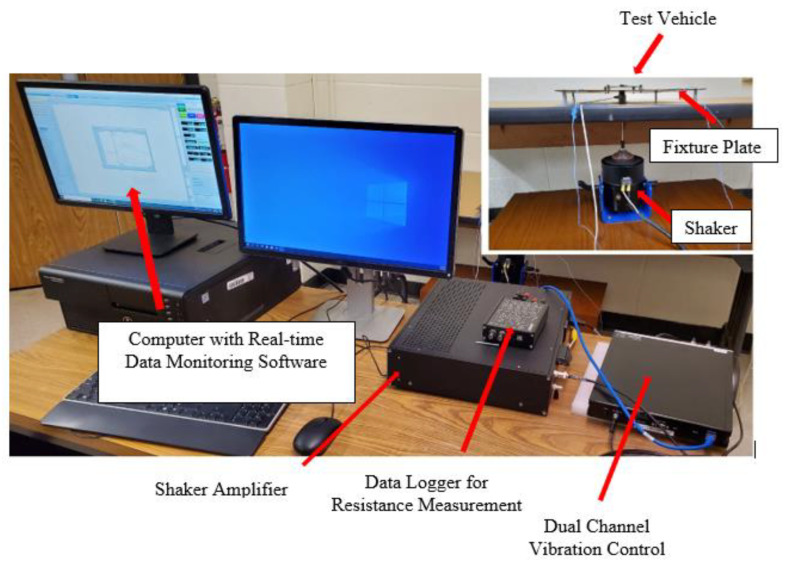
Detailed experimental setup.

**Figure 6:**
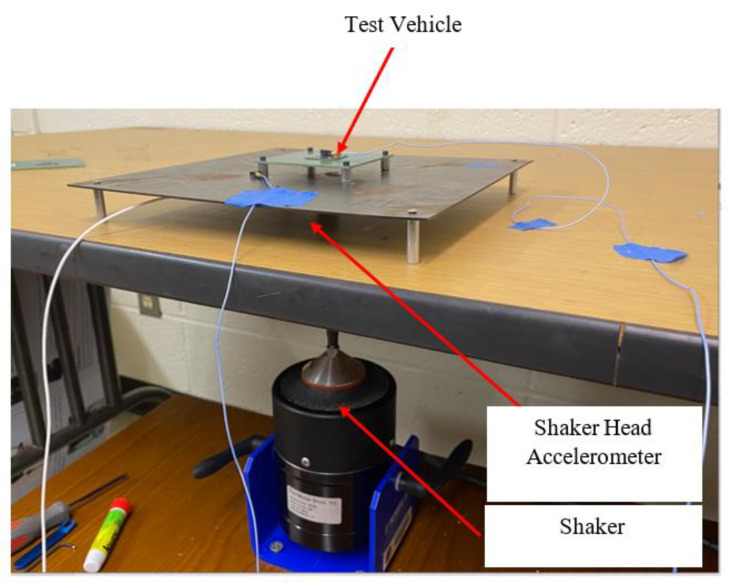
Close-up view of the shaker head accelerometer.

**Figure 7:**
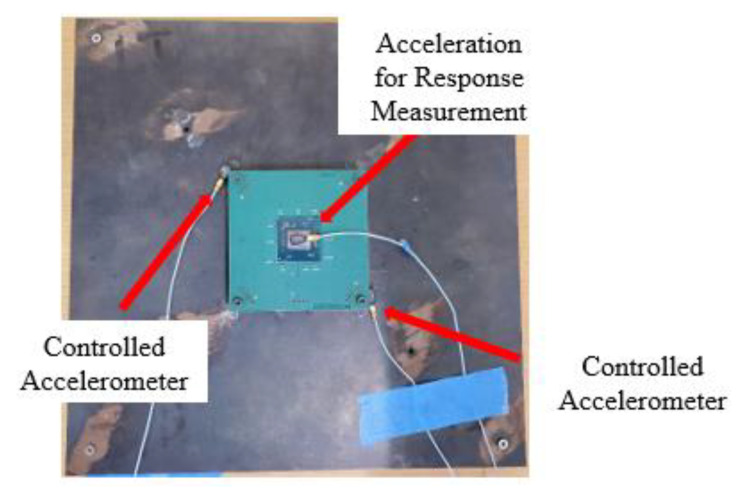
Detail of the test vehicle.

The experimental work for the prediction of the life of a solder joint addressed in this paper consisted of: (i) modal analysis of the test vehicle, (ii) a stepped sine test to validate the amplitude of vibration around the natural frequency of the test vehicle, and (iii) excitation of test vehicle at its first resonance frequency. In order to perform this experimental work, a suitable experimental setup needed to be designed. Figure 4 shows the details of the experimental setup. The setup consisted of: (i) an electrodynamic shaker from Modal Shop Inc., (ii) shaker amplifier, (iii) dual channel closed loop vibration controller (spider 80X), (iii) EDM vibration control system software from Crystal Instruments, (iv) data logger for resistance measurement from Data Translation, and (v) accelerometers for monitoring and controlling the signal at the desired locations. The traditional methods presented in the existing literature [8,10,11,12,15,19,20] utilize an electrodynamic shaker with a slip table assembly or fix the test vehicle directly to the shaker head. In contrast to the traditional methods of testing, the method presented in this work uses an electromagnetic shaker with a stringer rod. This rod works to dynamically decouple the shaker from the test fixture and the structure under testing. The stringer rod was equipped with an accelerometer at the top (head accelerometer), which was used to measure the direct input in the form of force/acceleration going to the fixture plate shown in Figure 5. A close-up view of the head accelerometer is shown in Figure 6. The head accelerometer was mounted exactly at the center of the fixture plate, such that the response at the four corner screws of the test vehicle will be identical. This is possible if the resonance vibration of the fixture is far away/below the excitation frequencies. Figure 7 shows the test vehicle with the fixture. The fixture was developed in such a way that (i) the resonance modes of the test vehicle do not interact with the resonance vibration of the fixture, (ii) there are no resonance frequencies of the fixture in the excitation frequency range. As shown in Figure 7, the test vehicle was mounted to the fixture using four corner screws and two corner accelerometers. These accelerometers were used to measure the signal at the corner screws and serve as a control excitation signal/input to the test vehicle. The response from the chip and the response of the head accelerometer were used as monitor responses. The equation of motion for the test vehicle shown in Figure 7 can be written as,

(1) $$\left[M\right]\left\{\ddot{U}\right\}+\left[K\right]\left\{U\right\}+\left[C\right]\left\{\dot{U}\right\}=-\left[M\right]\left\{l\right\}\left\{\ddot{Y}\right\}$$

where  [M], [K], and [C] respectively are the mass, stiffness, and the damping matrix of the test vehicle, {U} is the relative displacement of the test vehicle at the measured degrees of freedom with respect to the base/fixture motion, $\left\{l\right\}=cos\left\{\theta_{i}\right\}\;$ is the transformation vector with $\left\{\theta_{i}\right\}$ representing the angle between the *i^th^* degree of freedom and the direction of base motion, and $\left\{\ddot{Y}\right\}$ represents the controlled acceleration of the fixture plate at the four corner screws. Equation (1) can be solved using the conventional modal analysis technique to estimate the output response at the desired location, and modal damping ratios can be determined through direct experiments, which are used in the FE-based model to correlate the response between the experiments and the simulations. 

## 3. Results from Experiments and Simulations

In this section, the results from the experiments, correlated finite element model results, and the life predicted from the experiments and simulations for two samples of test vehicle are presented. Each sample consisted of two identically manufactured and assembled units of BGA Chips. As this paper mainly deals with the effect of board stiffening in the life of the solder ball, the detailed experimental and finite element study was carried out with two different thicknesses of PCB Board, as mentioned in Section 1.

### 3.1. Sine Sweep and Stepped Sine Testing

The purpose of sine sweep or frequency scan testing is to roughly estimate the natural frequency of the test vehicle. The natural frequency will then be further verified by the stepped sine testing. In order to perform a sine sweep test, the test vehicle shown in Figure 5, Figure 6 and Figure 7 was excited with a constant acceleration amplitude at a frequency ranging from (300–500) Hz at an octave rating of 0.5 octaves per minute for 1-mm thick board and (650–750) Hz for the 1.6-mm thick board. The maximum amplitude of vibration at each excitation frequency was recorded using the accelerometer glued to the top of the chip, as shown in Figure 7. For each sample, two identical PCBs with BGA chips were tested. Table 2 shows the natural frequency of the test vehicle obtained with the sine sweep test. It should be noted that for the same sample there was a slight difference in the natural frequency between board 1 and board 2. This inconsistency was possibly due to the manufacturing tolerance. The fatigue testing of each sample was carried out at the first mode, the most severe and likely bending mode for solder joint failure. The results for the higher modes of the test vehicles are not presented in this paper.

**Figure 8:**
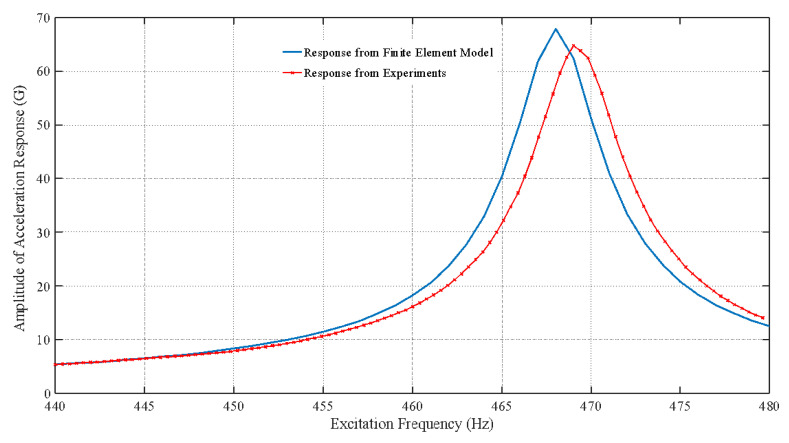
Frequency response function (FRF) of the test vehicle with a 1-mm board thickness.

**Figure 9:**
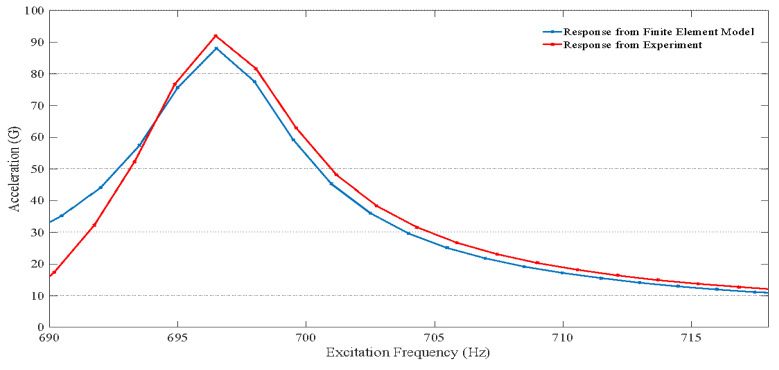
Frequency response function (FRF) of the test vehicle with a 1.6-mm board thickness.

**Figure 10:**
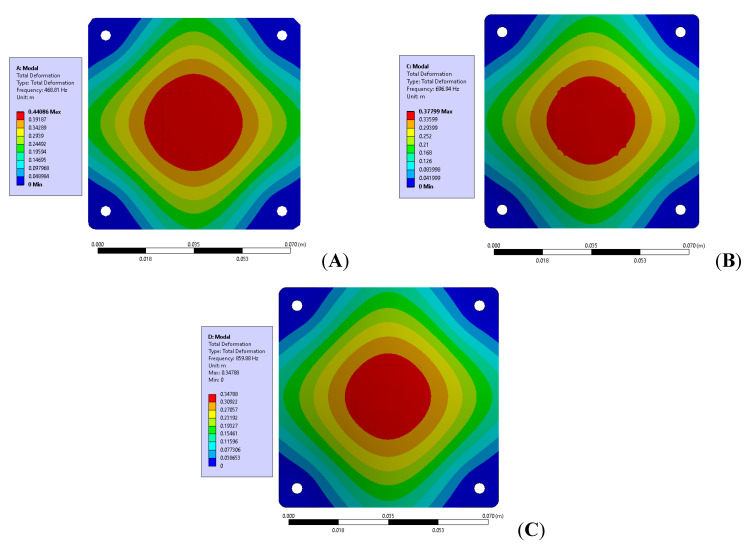
First mode response of the test vehicle obtained from simulation with different board thickness (**A**): 1 mm, (**B**): 1.6 mm, (**C**): 2 mm.

The next step of the experiment was to perform a stepped sine test to validate the natural frequency results and obtain the modal damping ratio for each sample of the test vehicle. In order to perform the stepped sine test, the test vehicle was excited with a constant amplitude of excitation in a narrow frequency band around the first mode natural frequency of the test vehicle, and at each excitation, the steady state amplitude of vibration was obtained. The frequency response function was created based on the steady state amplitude of vibration over the excited frequency range. The damping ratio was then estimated from the generated frequency response function, and the obtained damping ratio was used to calibrate the FE based model. The same procedure of stepped sine testing was carried out in the FE model, and the results obtained were compared with the experimental stepped sine test results. The half power bandwidth method was used to estimate the damping ratio of the test vehicle. Equation (2) shows the half power bandwidth equation to estimate the modal damping ratio.

(2) $$\xi =\frac{\Delta f}{2f_{n}}$$

where ξ represents the modal damping ratio, Δf is the difference in frequencies corresponding to the half power bandwidth (−3 dB) that is 0.707 of the peak amplitude, and $f_{n}$ corresponds to the frequency at the maximum amplitude of vibration. Figure 8 shows the comparison between the experimental and FE model stepped sine test results for sample 1 (1 mm thick board) for an excitation input of 0.5 G. As shown in Figure 8, the frequency response function (FRF) for the experimental and the FE simulations are very close to each other. The FRF is the data recorded by the accelerometer, which was at the top surface of the chip shown in Figure 7, whereas the FE model data corresponds to the nodal FRF at the top surface of the chip. As indicated by Figure 8, the first mode resonance frequency from the FE model was estimated to be 468.81 Hz, whereas the experimental value was found to be 469.2 Hz. Similarly, for sample 2 (1.6 mm thick board), the comparison of FRF between the experimental and FE model for an excitation input acceleration of 0.5 G is shown in Figure 9. From Figure 8 and Figure 9, it is clear that the increase in board thickness resulted in the increase in maximum amplitude of vibration at resonance and an increase in the resonance frequency of the test vehicle. Figure 10 shows the first bending mode vibration of the test vehicle with different PCB thicknesses extracted from the FE model.

### 3.2. Fatigue Test (FE Model) Results

**Figure 11:**
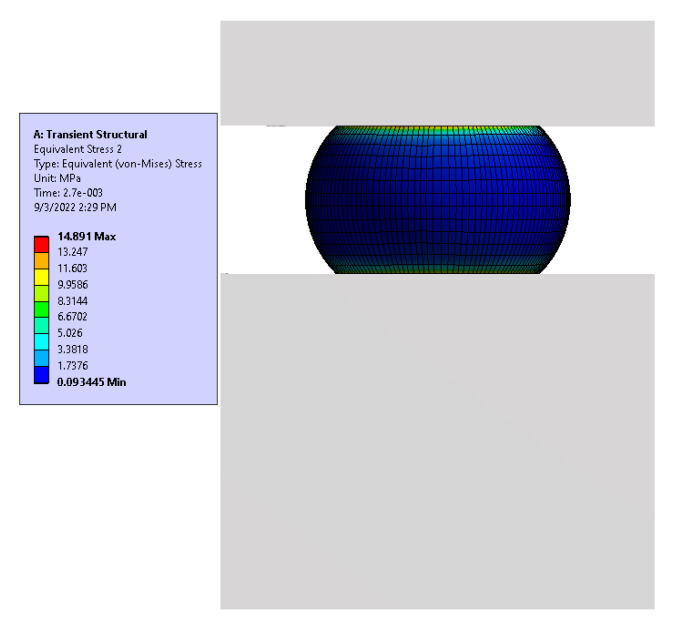
Maximum steady state stress amplitude at the solder ball (sample 1).

**Figure 12:**
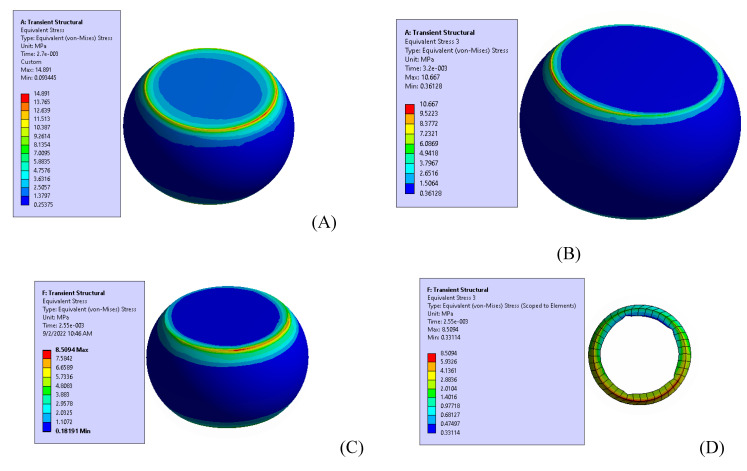
Maximum stress at the solder ball (**A**): 1 mm board, (**B**) 1.6 mm board, (**C**): 2 mm board, (**D**): elements for average stress.

The FE model of the test vehicle consisted of two parts: (i) a global model, and (ii) a local model. The acceleration responses of the test vehicle shown in Figure 8 and Figure 9 were verified from the experimental data using the global model of the system, and the displacement boundary conditions for the local model were obtained from the global model. In order to obtain the maximum displacement at the cut boundary, the global model was excited at the first mode resonance frequency at an excitation amplitude of 0.5 G. Since this paper deals with the effect of board thickness on the fatigue life of the solder ball, the same level of excitation (0.5 G) was used for both samples. There are several reasons for using the low input excitation amplitude including: (i) the test vehicle may not see excessive excitation amplitude at resonance frequencies, as the field vibration is random, (ii) there will be significant geometrical nonlinearities that will be excited when the test vehicle is subjected to higher excitation, (iii) the linear FE model results will not be valid and cannot be compared with experiments if significant nonlinearities are excited during experimentations, and (iv) there is a high probability of a change in the failure mechanism between the field and the laboratory based testing if the excitation amplitude is kept large. For example, the failure may be in the form of pad cratering, as opposed to solder joint failure. Figure 11 shows the maximum amplitude of the steady state stress at the solder ball. The maximum stress was at the top surface of the solder ball at the substrate/solder ball interface. Similarly, Figure 12 shows a comparison of the maximum steady state stress amplitude in the solder ball between sample 1 and sample 2, simulated using the FE based model. In addition, the effect of board stiffening in the stress induced in a solder joint was analyzed by adding a 2-mm board thickness. Clearly, the solder ball with the 1 mm thick PCB showed the maximum stress. As it is well known that the life of the solder joint is dependent on the maximum von Mises stress and that the von Mises stress is dependent upon the mesh density, the life of the solder ball was calculated using the volume average von Mises stress across the thin layer of solder elements [12,14,17,19]. The volume average von Mises stress was calculated using Equation (3).

(3) $$\sigma_{a}=\frac{\sum_{i=1}^{n}\sigma_{i}\times v_{i}}{\sum_{i=1}^{n}v_{i}}$$

where $\sigma_{i}$ is the maximum stress in the *i^th^* element, $v_{i}$ is the volume of the *i^th^* element, and *n* is the number of elements in a thin layer across the substrate solder interface. There is always a research question of how many thin layers of elements to take so that the accurate stress in the solder joint can be estimated [4,16,21,22]. Taking more layers will change slightly the stress value, which will affect the value of b and $\;\sigma_{f}$. Since the objective of this research was not the development of new parameters of b and $\;\sigma_{f}$ by varying the number of elements in the solder layer, only the elements containing the single thin layer of solder joint were taken into account, following the existing literature [21,22]. The life of the solder joint was estimated using the high cycle fatigue equation shown in Equation (4).

(4) $$\sigma_{a}=\sigma_{f}{\left(2N_{f}\right)}^{b}$$

where $\sigma_{f}$ is the fatigue strength coefficient, b is the fatigue strength exponent, and $2N_{f}$ is the number of cycles to failure. The material constant $\sigma_{f}$ and b can be estimated using the FE based simulated maximum stress. Several researchers obtained the material constant using the simulated maximum volume average von Mises stress [21,22,23]. Since our goal in this paper was to study the effect of board stiffness on the life of the solder joint (SAC 305), material constants were used from the existing literature. The material constants that were used to estimate the fatigue life of the solder joint using numerical simulation are tabulated in Table 3. Using Equations (3) and (4) and the material constant values, the fatigue life of the solder ball for two different samples with the changes in user PCB thickness are tabulated in Table 4.

### 3.3. Experimental Observation of Fatigue Life

The fatigue life of the solder joints in both samples were observed experimentally to verify the simulation results. In order to compare the results between the simulations and the experiments, the test vehicle was subjected to 0.5 G input acceleration at the four corner screws at the first mode resonance frequency. The failure of the solder joint was observed by monitoring the resistance increase of the test vehicle with a daisy chain assembly. The resistance of the corner balls was measured using a data logger, while the other ball resistances were monitored using a milliohm meter. In both samples of testing, the corner balls failed first, which agrees with the simulation results. As per the IEEE standard, an increase in resistance above 20% during testing is considered a failure. Table 5 shows the failure time for each sample and the comparison between the time predicted by FE simulation and experimentations. The percentage error between the simulation and the experimental results was around 20%, which is quite similar to the results reported in the existing literature [18,19,22,23]. In both simulations and experiments, the life of the solder ball was more than three-times higher for a stiffened PCB board. These results indicate that, even though the amplitude of acceleration increases as a result of board stiffening, the magnitude of the relative motion between the PCB and the package that is directly dependent on the frequency of excitation will decrease. The decrease in relative motion will decrease the corresponding von Mises stress experienced by the solder joint.

### 3.4. Failure Analysis

**Figure 13:**
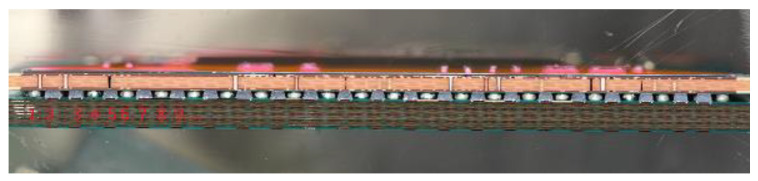
Cross-section of the package.

**Figure 14:**
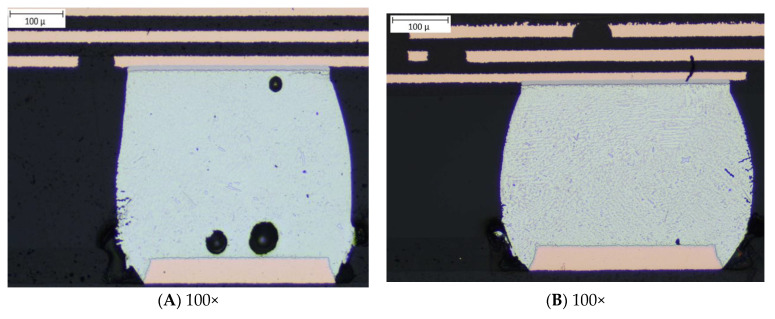

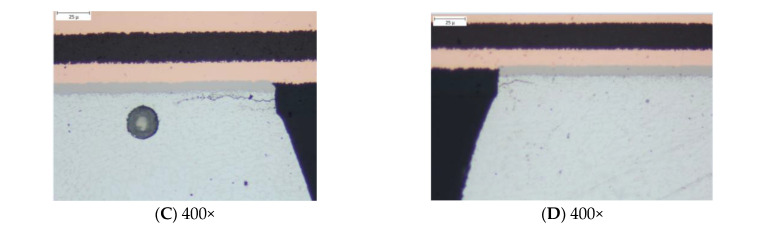
Optical microscopic images of the cracked solder ball: (**A**,**C**) 1.6-mm board, (**B**,**D**) 1-mm board.

**Figure 15:**
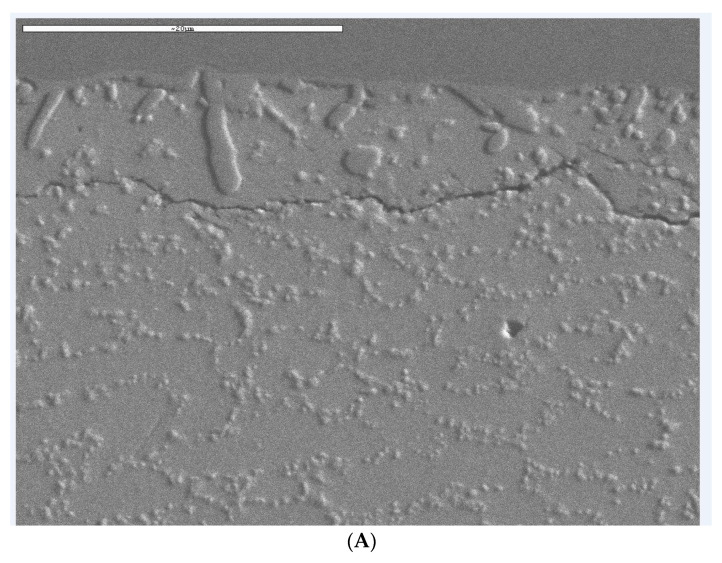

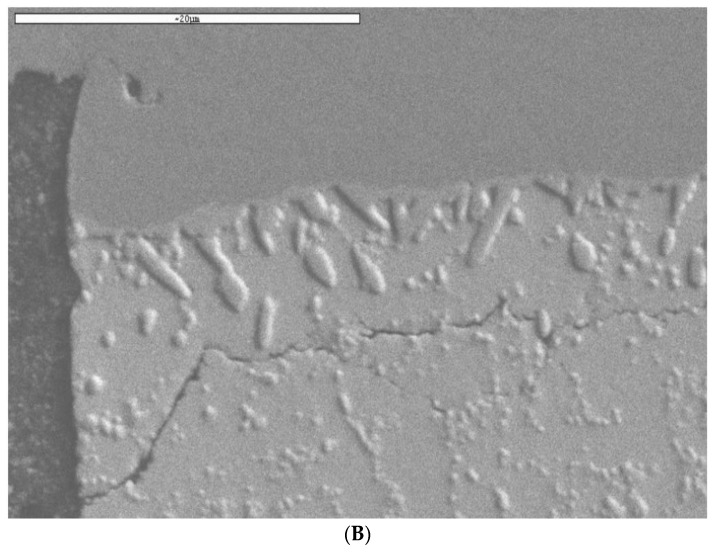
SEM Images of the cracked solder ball. (**A**) 2500×, 1.6 mm Board; (**B**) 2500×, 1 mm Board.

The failure of the solder joint was investigated using the cross-sectioning technique following the IPC-TM-650, 2.1.1. Figure 13 shows a cross-sectional view of the package. The failure mode was the crack in the bulk solder joint below the IMC layer in the package side. For each board tested, only one of the corner balls was found to fail and the failure mechanism was consistent. An interesting observation from the failure analysis was that there were no any failures at the IMC layer. Figure 14 shows an optical microscope image of the corner solder ball for the 1-mm and 1.6-mm board. As shown in Figure 14, the images (C) and (D) are the close-up view of (A) and (B). A scanning electron microscope (SEM) was used to further verify the crack of the bulk solder joint. Figure 15 shows the results taken from the SEM for the 1.6-mm board (A) and 1-mm (B). Images from SEM also further confirmed the bulk solder joint crack. The typical failure mode of the solder joint was brittle failure at the IMC layer, due to over-stressing (very high excitation) at the board. The thickness of the IMC layer and the roughness of the solder/IMC interface is associated with the migration of the failure mode [24,25]. With a higher excitation energy, the increased in IMC layer thickness decreased the tensile strength of the IMC, and at the same time, the reduction in the roughness of the interface reduced the stress concentration in the bulk solder in the vicinity of the interface [25]. These two factors caused the ductile fracture failure mode to migrate into the bulk solder near the interface to brittle failure in the IMC layer.

With the increase in excitation amplitude, the percentage of brittle failure in the IMC layer increased greatly. As reported in reference [25], under the strain rate of 2 s^−1^, the percentage of the brittle failure in the IMC layer increased to 80% which was the dominant failure mode of the solder joint. One of the possible reasons for this was that SAC 305 is sensitive to the strain rate, and during high amplitude excitation, the deformation localization in the bulk solder is suppressed, thus enhancing the brittle fracture in the IMC layer. On the other hand, at low excitation, the IMC layer thickness will not increase significantly, which results in the fracture in the bulk solder.

In this research, the board was excited at very low excitation, such that the dynamics of the test vehicle were completely in the linear range, which resulted in purely fatigue failure of the solder balls. Similar results of bulk solder cracking are reported in Reference [26], when the BGA package was subjected to harmonic excitation at room temperature.

## 4. Conclusions

In this paper, the effect of PCB board thickness on the life of a BGA solder joint was investigated using the resonance-based fatigue testing approach. The test vehicle consisted of two different samples of user PCB: sample 1 with a 1-mm thick user PCB board, and sample 2 with a 1.6-mm thick PCB board assembled to the BGA package. A finite element model using the local/global modeling approach was used to predict the life of the solder balls. A global model was utilized to verify the resonance frequency, and the maximum amplitude of vibration of the test vehicle was obtained using the stepped sine test approach. From the global model, the maximum amplitude of vibration was correlated with the direct experimental results, and the displacement results were obtained at the cut boundary. The displacement values obtained were used as a boundary condition for a local model, and a resonance based fatigue simulation was carried out in the local model. Similarly, first mode resonance-based fatigue testing was conducted on two sample test vehicles, by exciting the test vehicle at the first mode resonance frequency. The results from the numerical simulations and experimentations showed that the life of the solder ball increased significantly when the board was stiffened. Stiffening of the board increased the natural frequencies of the test vehicle and the increase in natural frequency reduced the maximum amplitude of the relative motion between the PCB and the package. The reduction in the amplitude of relative motion decreases the stress in the solder joint. The following conclusions are drawn from the study conducted with two samples: 

The life of a solder joint is directly dependent on the excitation frequency, therefore the aspect ratio of the user PCB plays an important role in defining the life of a solder ball.

FE based simulation results will agree well with the experimental results if an experiment is conducted with a low excitation input, such that the excitation will not induce system nonlinearities.

Future research should focus on finding out the effect of nonlinearities of the test vehicle in the life of a solder joint, as well as the failure mode of a solder joint with respect to different amplitudes of input excitation. 

## Figures and Tables

**Table 1:** Material Properties used in Finite Element Analysis.

SN	Materials	Young’s Modulus (GPa)	Poisson’s Ratio	Density (kg/m^3^)
1	PCB	15.6	0.39	1950
2	Solder Ball	51	0.3	7400
3	Substrate	15	0.35	1900
4	Silicon Chip	60	0.25	2330

**Table 2:** First Natural Frequency of the Test Vehicle.

Sample Number	Board Dimensions (mm)	Board 1 First Natural Frequency (Hz)	Board 2 First Natural Frequency (Hz)
Sample 1	77 × 77 × 1	469.2	459.76
Sample 2	77 × 77 × 1.6	696.58	699.3

**Table 3:** Material Constant Values for Fatigue Life Estimation.

Material Constant	Material Type	Numerical Values	Reference
$\sigma_{a}$(MPa)	SAC 305	64.8	[22]
b		−0.1443	

**Table 4:** SAC 305 Life Predicted through Simulation.

SN	PCB Dimensions	Solder Alloy	Input Excitation	Life to Failure
Sample 1	77 × 77 × 1	SAC 305	0.5 G	108 h, 27 min
Sample 2	77 × 77 × 1.6	SAC 305	0.5 G	450 h, 48 min

**Table 5:** Comparison of Experimental and Simulation Results.

Experimental Results				Simulation Results	% Error
Time to Failure			Mean Time to Failure	Time To Failure	
Sample 1	Test Vehicle 1	140 h, 26 min	137 h, 4 min	108 h, 27 min	20.87
	Test Vehicle 2	133 h, 38 min			
Sample 2	Test Vehicle 1	530 h, 19 min	540 h, 32 min	450 h, 48 min	16.60
	Test Vehicle 2	549 h, 43 min			

## Data Availability

The data presented in this study are available on request from the corresponding authors.
